# Supplementary material for: Metagenome-Assembled Viral Genomes Analysis Reveals Diversity and Infectivity of the RNA Virome of Gerbillinae Species
Source: Viruses. 2022 Feb 9;14(2):356. doi: 10.3390/v14020356 (PMC8874536; doi:10.3390/v14020356)
Supplement: Supplementary file 1 [file viruses-14-00356-s001.zip › Figures S1-S3.pdf]

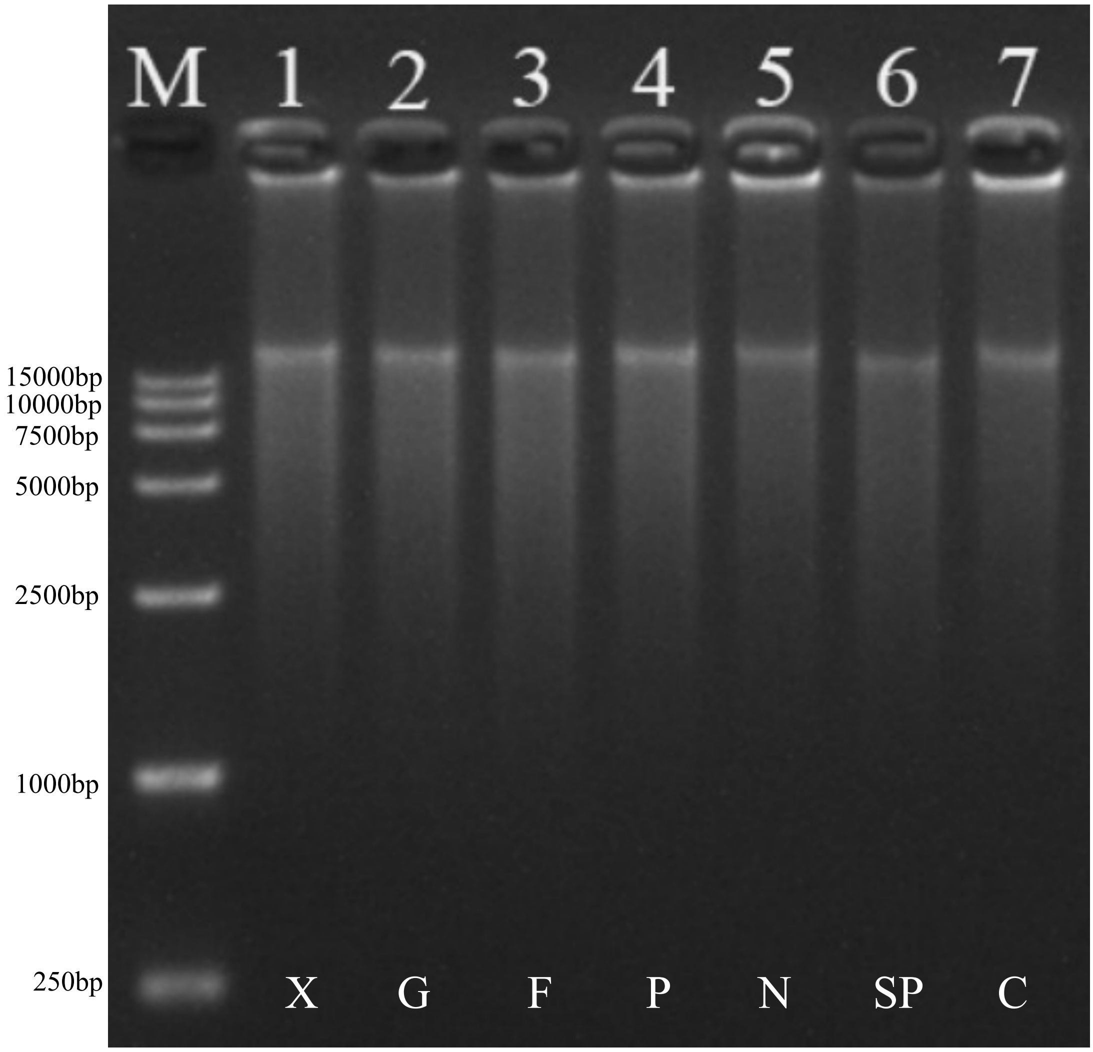

Figure S1. The electrophoresis figure of amplification products of 7 viscera pools, heart (X), liver (G), spleen (P), lung (F), kidney and bladder (SP), brain (N), intestines (duodenum, rectum, and cecum; C). (The sample size of DNA samples is 30ng; Marker is DL15000 with 2μL sample quantity.)

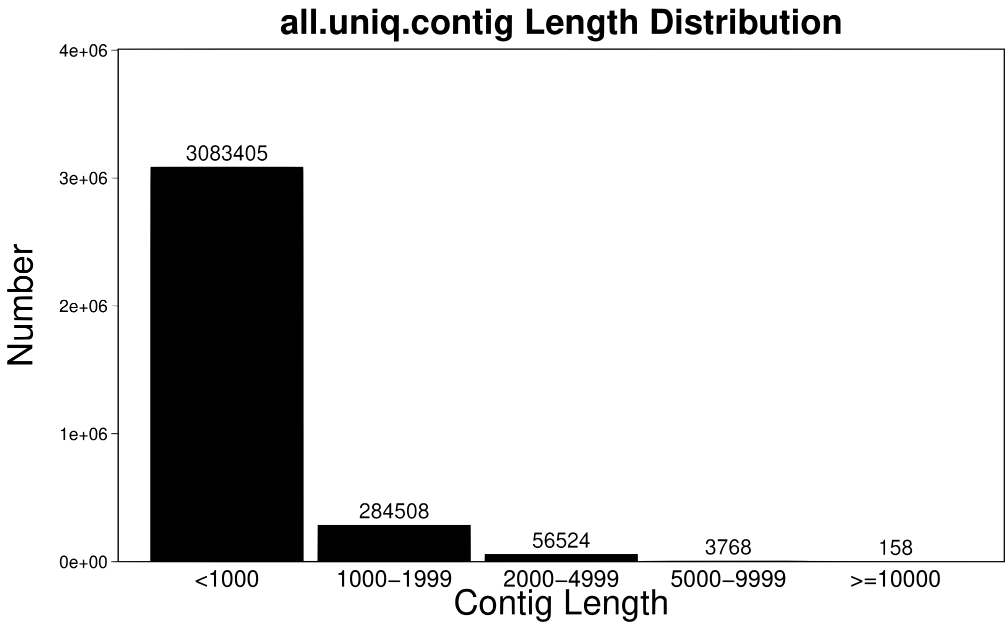

Figure S2. The length distribution map of all unique contig. (abscissa: the length of unique contigs, ordinate: the number of unique contigs.)

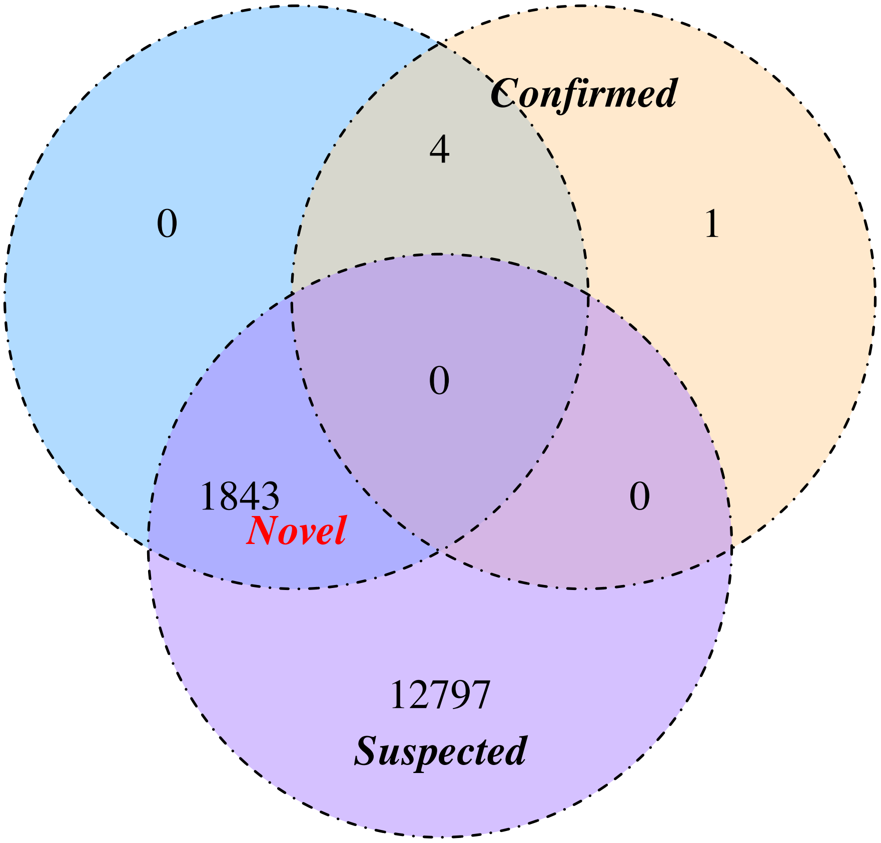

Figure S3. Comparison of the number of RNA viruses and unknown viruses contigs obtained by different strategies.
